# Efficacy of fungicides in controlling rice blast and dirty panicle diseases in Thailand

**DOI:** 10.1038/s41598-020-73222-w

**Published:** 2020-10-01

**Authors:** Nattapatphon Kongcharoen, Nipon Kaewsalong, Tida Dethoup

**Affiliations:** Department of Plant Pathology, Faculty of Agriculture, Kasetsart University, Bangkok, 10900 Thailand

**Keywords:** Microbiology, Plant sciences

## Abstract

In this study, the fungicidal activities of the fungicides azoxystrobin, difenoconazole + propiconazole, carbendazim, flutriafol, fluopyram + tebuconazole, mancozeb and thiophanate-methyl against rice blast and dirty panicle pathogens were evaluated under laboratory and field conditions. Mancozeb exhibited the highest level of fungicidal activity against the blast pathogen *Pyricularia oryzae*, with an EC_50_ value of 0.25 parts per million (ppm). The combination of two fungicides, fluopyram + tebuconazole, showed the strongest fungicidal effect against *Bipolaris oryzae* and *Curvularia lunata*, with EC_50_ values of 0.587 ppm and 0.435 ppm*,* respectively. Meanwhile, carbendazim and flutriafol demonstrated the best level of fungicidal activity against *Fusarium incarnatum*, with the lowest EC_50_ values of 0.211 ppm and 0.214 ppm*,* respectively. The results showed that the fungicides, triazole and strobilurin, had significant effects against rice blast and dirty panicle diseases. The combination of fluopyram + tebuconazole, when applied twice, was the most effective in reducing dirty panicle disease by up to 60% and increasing rice yield by 29% more than the untreated control. Fluopyram + tebuconazole, difenoconazole + propiconazole, flutriafol and azoxystrobin achieved stronger fungicidal activity against rice blast disease, reducing its severity by 32–33% when applied twice by foliar spraying. However, carbendazim, mancozeb and thiophanate-methyl had low to moderate fungicidal activity against both rice diseases in this study.

## Introduction

Rice is the most important economic crop in Thailand. The rice cultivation area is about 10,407,272 hectares: in 2018, approximately 32 million tons of rice production was directed to domestic consumption and export to world markets (https://www.fao.org/faostat/en/#data/QC/visualize). Rice diseases caused by fungi are considered the main constraint in rice production and cause both qualitative and quantitative losses^1,2^. In particular, rice blast disease caused by *Pyricularia oryzae* (*Magnaporthe grisea*) has been reported as the most significant disease, resulting in yield losses of up to 50%^1^. This fungus can infect rice at all growth stages, starting at the seedling stage, and causes severe damage to rice leaves. It can cause losses of up to 80% under favorable conditions (25–30 °C and 80–95% humidity) in susceptible rice cultivars, such as KDML 105 and RD 57 which are planted on half of Thailand’s total rice cultivation area^3,4^.

Dirty panicle disease or rice grain discoloration may be caused by many fungi, viz., *Alternaria padwickii* (Ganguly) M.B. Ellis; *Curvularia lunata* (Wakk) Boedjin; *Fusarium moniliforme* J. Sheld; and *Bipolaris oryzae* (Breda de Haan) Shoem^5^. Infection starts at the early boot stage and results in brown spots on rice hulls and the discoloration of rice grains. The germination of infected rice seeds is poor, and seedlings, when they emerge, are abnormal. Infected rice seeds are also the source of the inoculum of the pathogenic fungi, which is distributed through seed storage to new crops. This disease is a major cause of rice seed destruction and leads to losses in yield, both qualitatively and quantitatively, of up to 80%^6^. Dirty panicle disease has been reported in many countries where rice is a major crop, such as India, Pakistan, and Brazil^7–10^.

Although safe and eco-friendly approaches have been intensively promoted to combat rice diseases, the application of fungicides remains a widely used and effective approach for rice disease control^11–13^. Fungicides from many groups have been promoted in the Thai market for the control of rice diseases, especially single-site fungicides, such as triazole, strobilurin, dithiocarbamate, and antibiotics. Although single-site fungicides have proven to be very effective against various rice diseases, they have also been found to stimulate the development of fungicidal resistance among pathogen populations^14–16^. Many strategies have been developed to overcome this issue, including the synthesis of new fungicides^17–21^.

Rice pathogen resistance to various groups of fungicides has been reported in many countries^22,23^. Reports of the mutation of guanine to a G143A or a G143S in Qo of cytochrome b resulted in *M. oryzae* strain resistance to the Qo-inhibiting fungicide azoxystrobin^24^, while over-expression of the phosphatase gene *MoPTP2* in the Hog1p MAPK for osmoregulation revealed *M. oryzae* resistance to fludioxonil^25^.

The efficacy of fungicides in controlling rice diseases was investigated in the current study to determine the pathogens’ baseline sensitivity or resistance and the fungicides’ disease control potential under field conditions^12,13^. Azoxystrobin was reported to be more effective than propiconazole in controlling rice blast disease in the seedling stage in Australia^13^. Treating rice seeds with pyroquilon and azoxystrobin resulted in the greatest reduction in rice blast disease severity, whereas carboxin + thiram and tricyclazole significantly reduced fungi in seeds^11^.

In Thailand, studies have been conducted on many biological approaches to the control of rice diseases^6,26^. At the same time, formal reports on the efficacy of fungicides against rice diseases have not yet been published, even though fungicide application is the main method of rice disease management in Thailand. Thus, the aims of this study were to evaluate the fungicidal activity of fungicides against rice pathogenic fungi in vitro and to determine the efficacy of six major fungicides in controlling rice blast and dirty panicle diseases under field conditions.

## Results

### In vitro fungicide activity of fungicides against rice pathogens on PDA

The EC_50_ values of fungicides against the mycelial growth of the four major rice pathogens are shown in Table 1. Mancozeb exhibited the best fungicidal activity against the rice blast pathogen *P*. *oryzae*, with an EC_50_ value of 0.25 ppm. The combinations of fungicides showed strong fungicidal activity with, firstly, fluopyram + tebuconazole demonstrating the strongest fungicidal activity against *B. oryzae* and *C. lunata*, with EC_50_ values of 0.587 ppm and 0.435 ppm*,* respectively. Secondly, the other combination, flutriafol + carbendazim, showed the best fungicidal activity against *F. incarnatum*, with EC_50_ values of 0.211 ppm and 0.214 ppm*,* respectively.

**Table 1:** In vitro fungicidal activity of fungicides against rice pathogens on PDA.

Fungicide	EC_50_ values of fungicide (ppm)			
	*Bipolaris oryzae*	*Curvularia lunata*	*Fusarium incartanum*	*Pyricularia oryzae*
Azoxystrobin	125.872^d^	1,521.080^c^	94.105^e^	0.500^a^
Carbendazim	10.154^b^	483.157^b^	0.214^a^	21.250^b^
Difenoconazole + propiconazole	12.056^b^	0.871^a^	10.573^b^	0.500^a^
Fluopyram + tebuconazole	0.587^a^	0.435^a^	12.580^c^	0.500^a^
Flutriafol	5.632^ab^	5.587^a^	0.211^a^	0.500^a^
Mancozeb	10.058^b^	35.097^a^	224.076f.	0.250^a^
Thiophanate-methyl	30.586^c^	45.170^a^	25.301^d^	0.500^a^

Means followed by the same letter in the same column do not significantly differ at *p* < 0.05 when analyzed using Duncan’s test of one-way ANOVA.

### Effect of fungicides against dirty panicle disease in rice under field conditions

Dirty panicle disease in rice was less severe in 2017. The rainfall in 2016 was higher than in 2017, with the incidence of the disease in 2016 in untreated control being 83–89% (Table 2). In 2016, two applications of azoxystrobin showed a 60% reduction in dirty panicle disease, and when followed by fluopyram + tebuconazole or difenoconazole + propiconazole, applied twice, reduced the incidence of the disease by 56.66% and 52%, respectively.

**Table 2:** Effect of fungicides on dirty panicle disease under field conditions in 2016.

Treatment	Rate/L	No. of sprayings	% disease incidence	% pathogen incidence				Yield (kg)
				*Alternaria padwickii*	*Bipolaris oryzae*	*Curvularia lunata*	*Fusarium incarnatum*	
Azoxystrobin 25%SC	1 mL	1	54.33 ± 4.42^cd^	2.16 ± 0.17^b^	0.33 ± 0.00^a^	27.50 ± 2.31f.	28.16 ± 2.94^de^	2.86 ± 0.23^ab^
		2	29.33 ± 2.51^a^	0^a^	0^a^	5.00 ± 2.81^a^	7.16 ± 1.89^a^	3.32 ± 0.66^a^
Carbendazim 50%SC	0.6 mL	1	70.33 ± 6.71^e^	0^a^	0^a^	39.83 ± 3.02^i^	41.16 ± 4.57^hi^	2.67 ± 0.25^ab^
		2	55.50 ± 4.54^cd^	0^a^	0^a^	24.50 ± 3.17^e^	15.50 ± 1.08^bc^	3.09 ± 0.34^ab^
Difenoconazole + propiconazole 15% + 15%EC	0.75 mL	1	63.66 ± 5.02^de^	0^a^	0^a^	23.58 ± 2.10^e^	29.67 ± 3.21^de^	2.68 ± 0.36^ab^
		2	37.16 ± 2.57^ab^	0^a^	0^a^	10.67 ± 1.09^b^	16.83 ± 1.43^c^	3.01 ± 0.51^ab^
Fluopyram + tebuconazole 20% + 20%SC	1.2 mL	1	57.16 ± 4.65^d^	1.67 ± 0.52^b^	1.00 ± 0.00^ab^	19.16 ± 2.55^d^	27.50 ± 1.63^de^	2.71 ± 0.43^ab^
		2	32.50 ± 3.09^a^	0^a^	0^a^	3.00 ± 0.54^a^	11.00 ± 1.12^ab^	3.30 ± 0.17^a^
Flutriafol 12.5%SC	2 mL	1	74.33 ± 6.64^fg^	1.03 ± 0.71^ab^	0^a^	33.00 ± 1.47^h^	38.16 ± 2.54^gh^	2.77 ± 0.30^ab^
		2	42.16 ± 3.82^b^	0^a^	0^a^	4.00 ± 0.81^a^	36.00 ± 2.02^fg^	3.06 ± 0.19^ab^
Mancozeb 80%WP	2 g	1	70.50 ± 6.50^e^	4.83 ± 2.23^c^	2.33 ± 0.52^c^	40.83 ± 3.77^i^	42.00 ± 3.08^hi^	2.71 ± 0.25^ab^
		2	46.75 ± 5.38^bc^	1.66 ± 0.84^b^	1.33 ± 0.15^b^	13.50 ± 1.40^c^	32.50 ± 2.75^ef^	2.84 ± 0.22^ab^
Thiophanate-methyl 70%WP	1 g	1	74.33 ± 6.02^fg^	4.00 ± 0.41^c^	2.33 ± 0.57^c^	29.50 ± 2.12^g^	46.00 ± 3.14^ij^	2.68 ± 0.28^ab^
		2	43.00 ± 3.52^b^	0^a^	0^a^	19.67 ± 1.64^d^	24.67 ± 2.17^d^	3.04 ± 0.36^ab^
Water (control)		1	83.33 ± 7.06^gh^	8.67 ± 1.01^d^	3.83 ± 0.16^d^	57.67 ± 4.19^j^	48.83 ± 4.56^j^	2.57 ± 0.22^b^
		2	89.16 ± 8.44^h^	8.15 ± 1.62^d^	3.69 ± 0.38^d^	56.33 ± 3.24^j^	43.00 ± 4.04^hi^	2.54 ± 0.36^b^

Means ± standard derivations followed by the same letter in the same column do not significantly differ at *p* < 0.05, when analyzed using Duncan’s test of one-way ANOVA.

However, all fungicides tested in this study showed low effectiveness against dirty panicle disease when applied once, with less than 30% reduction in the severity of the disease. The main plant pathogenic fungi causing dirty panicle disease in this study were *C. lunata* (56–57%) and *F. incarnatum* (43–48%). The applications of all fungicides reduced the occurrence of pathogens on the treated rice seeds. The application of fluopyram + tebuconazole showed the greatest reduction in *C. lunata* (up to 53%), whereas azoxystrobin, when applied twice, exhibited the best suppression of *F. incarnatum* (36%).

Rice yields were significantly higher in rice exposed to fungicide treatments than in the untreated control. The maximum yield increases were found when the fungicide was applied twice, with the treatments using azoxystrobin and fluopyram + tebuconazole increasing rice yield by 30.71% and 29.92%, respectively, when compared to the control.

In 2017, the dirty panicle disease incidence was 62.83–69.05% in untreated controls, which was significantly lower than in 2016 as the rainfall in 2017 was less than in 2016. However, the fungicidal effect results of the fungicides against this disease in 2017 corresponded to the results in 2016. Two applications of all the fungicides resulted in better disease suppression than only one application. Treatments of fluopyram + tebuconazole and azoxystrobin, when applied twice, showed the best disease reduction of 52.22% and 50.89%, respectively. Moreover, two applications of difenoconazole + propiconazole and flutriafol reduced the disease incidence by 45%. One application of the fungicides showed low to moderate disease reduction activity of 29–20% (Table 3).

**Table 3:** Effect of fungicides against dirty panicle disease under field conditions in 2017.

Fungicide	Rate/L	No. of sprayings	% disease incidence	% pathogen incidence				Yield (kg)
				*Alternaria padwickii*	*Bipolaris oryzae*	*Curvularia lunata*	*Fusarium incarnatum*	
Azoxystrobin 25%SC	1 mL	1	38.33 ± 1.44^cde^	0.33 ± 0.07^a^	0^a^	21.33 ± 1.48^bc^	18.00 ± 1.73^d^	3.29 ± 0.50^a^
		2	18.16 ± 2.81^a^	0.33 ± 0.07^a^	0^a^	14.33 ± 0.82^a^	14.33 ± 1.04^c^	3.62 ± 0.29^a^
Carbendazim 50%SC	0.6 mL	1	40.16 ± 1.52^de^	0.33 ± 0.07^a^	1.02 ± 0.23^b^	26.00 ± 2.07^d^	13.83 ± 1.25^c^	3.04 ± 0.65^a^
		2	33.16 ± 2.51^c^	0.66 ± 0.12^a^	0.33 ± 0.07^a^	19.17 ± 2.46^b^	10.00 ± 1.17^b^	3.13 ± 0.58^a^
Difenoconazole + propiconazole 15% + 15%EC	0.75 mL	1	35.83 ± 3.48^cd^	1.00 ± 0.00^ab^	0.33 ± 0.07^a^	19.50 ± 2.12^bc^	19.83 ± 2.13^de^	3.15 ± 0.37^a^
		2	24.00 ± 0.86^b^	0.33 ± 0.07^a^	1.50 ± 0.16^c^	12.17 ± 2.89^a^	13.50 ± 2.02^c^	3.34 ± 0.48^a^
Fluopyram + tebuconazole 20% + 20%SC	1.2 mL	1	33.00 ± 2.59^c^	1.33 ± 0.31^b^	0^a^	21.00 ± 1.76^bc^	11.67 ± 1.14^bc^	3.21 ± 0.40^a^
		2	16.81 ± 3.07^a^	0.56 ± 0.12^a^	0^a^	11.50 ± 1.19^a^	6.00 ± 1.06^a^	3.66 ± 0.15^a^
Flutriafol 12.5%SC	2 mL	1	39.03 ± 3.11^cde^	0^a^	0^a^	25.33 ± 2.04^d^	21.58 ± 1.37^e^	3.22 ± 0.61^a^
		2	23.83 ± 2.25^b^	0.33 ± 0.07^a^	0^a^	13.50 ± 1.17^a^	12.17 ± 0.54^bc^	3.35 ± 0.39^a^
Mancozeb 80%WP	2 g	1	43.58 ± 3.29^e^	0^a^	1.66 ± 0.52^c^	30.33 ± 2.51^e^	18.17 ± 2.82^d^	3.01 ± 0.44^a^
		2	33.66 ± 3.61^c^	0^a^	1.39 ± 0.57^bc^	22.17 ± 1.03^bcd^	19.33 ± 2.08^de^	3.08 ± 0.16^a^
Thiophanate-methyl 70%WP	1 g	1	42.33 ± 2.61^e^	1.33 ± 0.31^b^	0^a^	30.67 ± 2.61^e^	9.17 ± 1.35^b^	3.00 ± 0.27^a^
		2	36.16 ± 1.52^cd^	0.33 ± 0.07^a^	0^a^	23.33 ± 2.04^cd^	12.17 ± 1.03^bc^	3.15 ± 0.53^a^
Water (control)		1	62.83 ± 5.25^f^	2.35 ± 0.66^c^	2.73 ± 0.41^e^	35.17 ± 3.00^f^	31.50 ± 3.21^f^	2.89 ± 0.41^a^
		2	69.05 ± 6.73^g^	2.76 ± 0.25^d^	2.32 ± 0.37^de^	40.21 ± 3.81^g^	35.00 ± 3.03^g^	2.92 ± 0.55^a^

Means ± standard derivations followed by the same letter in the same column do not significantly differ at *p* < 0.05, when analyzed using Duncan’s test of one-way ANOVA.

*Curvularia lunata* and *F. incarnatum* were also the predominant fungi found in diseased rice seeds in 2017. They were significantly reduced when fluopyram + tebuconazole and azoxystrobin were applied twice. Corresponding to their potent fungicidal activity, two applications of fluopyram + tebuconazole and azoxystrobin resulted in increases in rice yield weight by 25.34% and 23.97%, respectively, compared to the control treatment.

### Effects of fungicides against rice blast disease under field conditions

The performance of fungicides in controlling rice blast disease caused by *P. oryzae* under field conditions is presented in Table 4. Two applications of all fungicides tested in this study were more effective in reducing disease incidence than one application. Difenoconazole + propiconazole, flutriafol, azoxystrobin, and fluopyram + tebuconazole had no significant fungicidal activity against rice blast disease. The best reduction in rice blast disease incidence ranging from 32.84 to 33.25% was obtained when these fungicides were applied once.

**Table 4:** Effects of fungicides against rice blast disease under field conditions.

Fungicide	Rate/L	% disease incidence	
		One application	Two applications
Azoxystrobin 25%SC	1.10 mL	8.14 ± 0.24^a^	3.33 ± 0.09^a^
Carbendazim 50%SC	0.75 mL	16.23 ± 1.06^b^	9.04 ± 0.41^c^
Difenoconazole + propiconazole 15% + 15%EC	0.75 mL	7.86 ± 0.35^a^	3.02 ± 0.22^a^
Fluopyram + tebuconazole 20% + 20%SC	1.2 mL	8.27 ± 0.26^a^	4.62 ± 0.25^ab^
Flutriafol 12.5%SC	2 mL	8.07 ± 0.19^a^	4.13 ± 0.16^ab^
Mancozeb 80%WP	2.25 g	18.51 ± 1.10^c^	10.68 ± 0.67^c^
Thiophanate-methyl 70%WP	1.2 g	9.25 ± 0.51^a^	5.75 ± 0.40^b^
Water (control)		41.11 ± 2.84^d^	45.18 ± 2.43^d^

Means ± standard derivations followed by the same letter in the same column do not significantly differ at *p* < 0.05, when analyzed using Duncan’s test of one-way ANOVA.

Amongst the tested fungicides, thiophanate-methyl showed moderate fungicidal activity against rice blast disease, whereas mancozeb and carbendazim, even when applied twice, displayed low levels of activity against the disease**.**

## Discussion

Fungicide application has been the effective option for controlling rice diseases in the crops of Thai farmers. In the laboratory tests, the broad spectrum fungicide, mancozeb, showed the best fungicidal activity among the eight fungicides in inhibiting *P. oryzae*, with the lowest EC_50_ value of 0.206 ppm. However, mancozeb showed a low level of activity against both rice blast and dirty panicle diseases in field tests when compared to the other fungicides (Tables 2, 3, 4). Mancozeb is an effective fungicide which carries out the protective activity of inhibiting spore germination but only on leaf surfaces^27^. Rainfall may have lowered mancozeb’s fungicidal activity against rice diseases in field trials^28,29^.

Dirty panicle or seed discoloration is a serious rice disease in Thailand. This disease has shown greater severity under conditions of high rainfall and humidity^29^, evidenced by the disease’s higher incidence in 2016 than in 2017. Biological control agents viz. *Trichoderma*, *Talaromyces*, and *Bacillus* against this disease have been reported in Thailand^6,26,29^. In the current study, the combined fungicides of triazole and fluopyram + tebuconazole demonstrated dirty panicle disease suppression of up to 60% when applied twice, while two applications of the strobilurin fungicide, azoxystrobin, reduced the incidence of the disease by 56% in 2016, a year of heavy rainfall. This corresponds to previous studies which reported the potential of strobilurin and triazole fungicides for controlling seed-borne diseases of rice. What was apparent in the current study was that rice treated with a mixture of azoxystrobin + difenoconazole led to considerable reductions of *Fusarium* sp., *Gerlachia* sp., and *Bipolaris* sp. infections on rice seeds after harvesting^30^. Recently, a study reported that tebuconazole was superior in controlling rice grain discoloration followed by propiconazole, trifloxystrobin + tebuconazole, trifloxystrobin, and tricyclazole^8^.

Moreover, the effects of the tested fungicides in controlling rice blast disease caused by *P. oryzae* under field conditions (Table 3) showed that the strobilurin fungicide, azoxystrobin, and the mixed triazole fungicides, difenoconazole + propiconazole and fluopyram + tebuconazole, exerted greater potential effect in rice blast disease reduction, whereas mancozeb showed the lowest level of fungicidal activity. Various researchers have reported that propiconazole (0.1%), azoxystrobin + difenoconazole (0.1%), and floxystrobin + tubuconazole (0.04%) were found to have significant effects on controlling rice blast disease, reducing the disease by 60.3%, 55.1%, and 53.3%, respectively^31^. Meanwhile, tricyclazole and mancozeb were found to have the greatest and the least levels of fungicide activity against rice blast disease at 67.9% and 5.5%, respectively. One study reported the efficacy of 50% carbendazim in controlling rice blast disease was 59–61%^32^, but the fungicide displayed a more moderate fungicidal effect in controlling this disease in the current study with disease reduction of 35.8%.

In the in vitro tests, the EC_50_ values of azoxystrobin and propiconazole against *P. oryzae* were reported in the range of 0.006–2.02 ug/mL and 0.06–0.91 ug/mL, respectively^13,33^. The EC_50_ values of azoxystrobin and propiconazole against this fungus were both 0.5 ug/mL. This result indicated that the Thai strain of *P. oryzae* was still tending to be sensitive to both fungicides.

The efficacies of other fungicides in the triazole group in controlling rice diseases have also been reported. The effects of epoxiconazole and tricyclazole reduced rice blast disease by 75–77% and 73–76%, respectively^32^. Rice treated with tricyclazole showed significantly greater resistance to rice blast disease, as well as increases in the number of tillers/plant, the number of spikelets/panicle, panicle length, grain yield, and seed weight^34^. Moreover, propiconazole (0.1%) and kresoxim methyl (0.1%) were reported to reduce false smut disease in rice by 22.57% and 19.79%, respectively^35^.

The triazole and strobilurin fungicides demonstrated significant fungicidal effects against both rice blast and dirty panicle diseases. These systemic fungicides are single-site inhibitors. In terms of modes of action, the triazole fungicidal group inhibits sterol biosynthesis in membranes by interrupting C14-demethylase activity, while strobilurin inhibits enzyme activities in mitochondrial respiration^36^. However, both fungicidal groups have been considered as high-risk fungicides, while *P. oryzae* has been studied as a highly destructive pathogen by the Fungicide Resistance Action Committee^24,37^. Thus, it has been recommended that these fungicides should be applied in rotation and mixed with fungicides from groups having other modes of action or with low-risk fungicides to mitigate the development of fungicide resistance in the pathogen population.

The triazole and strobilurin fungicides showed significant effects in controlling dirty panicle and rice blast diseases under field conditions. Among the fungicides tested in this study, the combined fungicides of fluopyram + tebuconazole was observed to be the most effective, reducing dirty panicle disease by up to 60% and, when applied twice, increasing rice yield over the untreated control. Fluopyram + tebuconazole, difenoconazole + propiconazole, and azoxystrobin, when applied twice, revealed stronger fungicidal effects against rice blast disease with reductions ranging from 32.84 to 33.25%. On the other hand, mancozeb was found to have the lowest level of fungicide activity against both rice diseases in field trials.

## Methods

### In vitro fungicide activity of fungicides against rice pathogens on PDA

The in vitro evaluation of fungicides against four rice pathogens (Table 1) was conducted using the dilution plate method, as described in prior research^6^. Three strains of each rice pathogen were isolated from diseased rice plants and identified by molecular techniques. Primer pairs, ITS1 and ITS4 were used for ITS gene amplification^38^ and their gene sequences were submitted to GenBank numbers MG914427-MG914428, MG914430, and MT796346-MT796354. Each fungicide was dissolved in distilled water, mixed with 9 mL of warm potato dextrose agar (PDA) medium and then poured into Petri dishes to obtain final concentrations of 100 ppm, 1000 ppm, and 10,000 ppm in separate Petri dishes. A mycelial plug (5 mm in diameter) of each pathogen, obtained from a 7-day-old colony, was cut with a sterile steel borer and kept in the center of PDA plates containing a fungicide at each concentration. The plates were incubated at room temperature for 14 days. A PDA plate without fungicide served as the negative control. Inhibition levels were calculated using the formula:

$$\left[ {\left( {x - y} \right)/x} \right] \, \times { 1}00$$

where *x* = colony radius of the plant pathogenic fungus in the negative control, *y* = colony radius of plant pathogenic fungus in the presence of the tested fungicide.

Each treatment was performed with five replications and repeated three times independently. The EC_50_ value of each fungicide was calculated by probit analysis.

### Effects of fungicides against dirty panicle disease under field conditions

The efficacy of fungicides against dirty panicle disease under field conditions has been previously described^39^. The experiment was conducted in a lowland field in Khok Samrong district, Lopburi province in central Thailand. Each plot was 4 m × 3 m in size with 0.30 m between rows and a total of 13 rows per plot. Each row consisted of 13 hills with five rice seedlings (cv. KDML 105) per hill and 0.30 m distance between hills. The distance between plots was 1.5 m, and the plots were in a completely randomized design. Conventional cultivation practices were applied.

The rice plants were sprayed with each fungicide at the recommended rate, with water applied as the negative control (Table 2). The first treatment was applied at the early boot stage, while the second treatment was applied 4 days after the first. Rice panicles were sprayed with 3 L of each fungicide (or water for the control) per plot with separate portable sprayers, and each treatment consisted of five plots (replicates). When the rice panicles were aged 40 days, the treated rice panicles of each treatment were hand harvested and dried in the shade for 3 days.

Infection by disease pathogens was observed using the International Seed Testing Association^40^ method. Two hundred dried rice seeds from each treatment were randomly selected and placed in Petri dishes containing a water-soaked blotter (25 seeds per dish). They were then incubated for 7 days at room temperature under a cycle of 12 h of light and 12 h of darkness. The pathogens of dirty panicle disease were observed on the rice seeds using a stereo microscope (Olympus SZ51) and identified based on their morphological characteristics. Their incidence in each treatment was recorded. The experiment was conducted in two successive years, 2016 and 2017, during the July–November period.

### Effects of fungicides against rice blast disease under field conditions

Field experiments under natural infection were conducted at Bang Bua Thong district, Nonthaburi province in central Thailand. The distance between plots was 1 m. Each row consisted of 13 hills with 7-day-old rice seedlings (cv. KDML 105) per hill. When rice blast disease symptoms appeared in 5–10% of the plants under natural infection at the tillering stage, the rice plants were treated for the first time with the tested fungicides at the recommended rate, and water was applied as a negative control. Two applications were made, with rice leaves treated the second time 10 days after the first application. The rice plants were sprayed with 3 L of each fungicide (or water for the control) per plot with separate portable sprayers, and each treatment consisted of five plots (replicates).

Seven days after each application, a total of 30 s leaves of the treated rice plants from each plot were hand harvested to assess disease incidence. Disease incidence was recorded in accordance with the scoring scale (0–9) presented by the International Rice Research Institute^41^. The percentage of disease incidence was analyzed using the formula^42^:

$$[{\text{S}}({\text{r}} \times {\text{nr}})/{9} \times {\text{Nr}}] \times {1}00$$

where *r* = rating value (0–9); *nr* = number of infected leaves with a rating of *r*; and *Nr* = total number of leaves tested in each replication.

The experiments were conducted during July and August in two successive years, 2017 and 2018.

### Statistical analysis

The results for fungicides tested in this study against dirty panicle disease in rice in 2016 differed significantly from those in 2017, although no significant difference occurred between the repetitions of each experiment. Thus, the data from the replicate experiments in each year were pooled and analyzed separately for 2016 and 2017. No significant differences were found between the repetitions of each experiment in testing for the control of rice blast disease, with the separate data from each experiment pooled and analyzed. The data were submitted to the analysis of variance (ANOVA), and means were compared with Duncan’s multiple range test (*p* < 0.05), using the statistical program SPSS (v.19) (IBM Corporation, Somers, NY).

### Ethical approval

This article does not contain any studies involving human participants or animals performed by any of the authors.
